# Do the rat anterior thalamic nuclei contribute to behavioural flexibility?

**DOI:** 10.1016/j.bbr.2018.10.012

**Published:** 2019-02-01

**Authors:** Lisa Kinnavane, Eman Amin, John P. Aggleton, Andrew J.D. Nelson

**Affiliations:** School of Psychology, Cardiff University, Cardiff, CF10 3AT, UK

**Keywords:** Anterior thalamic nuclei, Discrimination learning, Executive function, Response conflict, Reversal, Switching

## Abstract

The rodent anterior thalamic nuclei (ATN) are vital for spatial memory. A consideration of their extensive frontal connections suggests that these nuclei may also subserve non-spatial functions. The current experiments explored the importance of the ATN for different aspects of behavioural flexibility, including their contribution to tasks typically associated with frontal cortex. In Experiment 1, rats with ATN lesions were tested on a series of response and visual discriminations in an operant box and, subsequently, in a water tank. The tasks included assessments of reversal learning as well switches between each discrimination dimension. Results revealed a mild and transient deficit on the operant task that was not specific to any stage of the procedure. In the water tank, the lesion animals were impaired on the reversal of a spatial discrimination but did not differ from controls on any other measure. Experiment 2 examined the impact of ATN damage on a rodent analogue of the ‘Stroop’, which assesses response choice during stimulus conflict. The lesion animals successfully acquired this task and were able to use contextual information to disambiguate conflicting cue information. However, responding during the initial presentation of conflicting cue information was affected by the lesion. Taken together, these results suggest that the ATN are not required for aspects of behavioural flexibility (discrimination learning, reversals or high-order switches) typically associated with the rat medial prefrontal cortex. The results from Experiment 2 suggest that the non-spatial functions of the ATN may be more aligned with those of the anterior cingulate cortex.

## Highlights

• Anterior Thalamic Nuclei (ATN) lesions produce mild nonselective effects on a strategy shift task.
• Spatial, but not non-spatial, reversals affected by ATN lesions.
• ATN lesions impair initial choice performance during conflicting cue information.
• ATN lesions do not reproduce the effects of prelimbic damage.
• ATN may be functionally aligned with the anterior cingulate cortex.

## 1 Introduction

The rodent anterior thalamic nuclei (ATN) function in close association with the hippocampus to support spatial learning and navigation [[1], [2], [3]]. In addition to their dense hippocampal connections [[4], [5], [6]], the ATN are reciprocally connected with many frontal areas, including the prelimbic, anterior cingulate and orbitofrontal cortices [[7], [8], [9], [10]]. These frontal interconnections suggest that the ATN may have additional cognitive functions beyond the spatial domain. Preliminary support for this proposition comes from clinical evidence of executive dysfunction in patients with damage in the anterior thalamus [11,12]. Other evidence comes from the demonstration that the rodent ATN are required for recency judgments, an ability closely aligned to hippocampal-frontal interactions [[13], [14], [15], [16], [17], [18]]. Recent work has also uncovered a role for the ATN in attentional control: rats with ATN lesions were slower to acquire new discriminations that involved the previous stimulus dimension (intradimensional shifts), while outperforming control rats when learning new discriminations that involved previously irrelevant stimulus dimensions (extradimensional shifts) [19].

Behavioural flexibility, or the ability to update responding as environmental contingencies change, is a key executive function mediated by the rodent prefrontal cortex [20,21]. However, behavioural flexibility encompasses a range of different cognitive processes that are in-turn supported by diverse frontal, corticostriatal and corticothalamic systems [[22], [23], [24], [25]]. For example, reversal learning by rats is sensitive to orbitofrontal cortex damage [[26], [27], [28], [29]], while the ability to switch between different stimulus dimensions or response strategies, as well as the use of high-order rules to guide goal-directed behaviour, depend on the integrity of the medial prefrontal cortex and in particular prelimbic cortex [[30], [31], [32], [33], [34], [35]]. The current set of experiments, therefore, sought to explore systematically the potential involvement of the rat ATN in these different processes and to characterise further the contribution these thalamic nuclei to tasks that are typically associated with these diverse frontal areas.

Initially, the present study examined reinforced T-maze alternation to test the effectiveness of the ATN surgeries (Experiment 1A). Next, Experiment 1B and 1C examined the impact of ATN lesions on both visual and response discriminations, where the latter stimuli differed in their egocentric position with respect to the animal (right or left). Two types of discrimination problem were included in the study: reversals within the same stimulus class and switching from one stimulus class to another (e.g., from response to visual). Experiment 1B used an automated chamber to measure the ability to switch between visual-based and response-based discriminations, as well as reversals within each stimulus class. The visual stimuli consisted of the lights in the test chamber while the response discrimination involved pressing either the right or left lever in the chamber. Previous research with rats has shown that medial prefrontal cortex manipulations impair the ability to switch between these visual and response-based discriminations [31,32]. In Experiment 1C the response requirements were changed so rats were first trained in a water-tank to navigate towards the right or left choice arm, then reverse, and then switch from the response-based egocentric discriminations (swim right or left) to a visual discrimination (swim to the black or white cue).

The final experiment, involving a different cohort of rats, assessed the impact of ATN damage on a rat analogue of the Stroop task [34,36]. In this task, rats concurrently learn two conditional discriminations, one visual and one auditory, in two distinct contexts (Experiment 2). Each animal acquires four distinct instrumental contingencies. At test, animals receive compound audiovisual stimuli either composed of those stimulus elements that had elicited the same response (‘congruent’ trials) or different responses (‘incongruent’ trials) during training. Normal animals use contextual information to disambiguate the conflicting information provided by incongruent trials [37]. This task, therefore, assesses behavioural flexibility in response to conflict as well as the use of higher order rules to guide instrumental behaviour; processes that depend on the anterior cingulate and prelimbic cortices respectively [34,38,39].

## 2 Experiment 1: strategy shifts and reversals

### 2.1 Materials and methods

#### 2.1.1 Animals

Experiment 1 used 30 naive, male Lister Hooded rats (*Rattus norvegicus*) supplied by Envigo (Bicester, United Kingdom). The rats were housed in groups of three or four under a 12-hour light/dark cycle. All testing occurred during the light cycle. The animals had free access to water but were restricted to 85% of their free-feeding weight for the duration of the experiments, with the exception of the water-tank task (Experiment 1C) when food was available *ad libitum*. All animals received repeated handling before the start of the first experiment. The experiments were in accordance with the UK Animals (Scientific Procedures) Act (1986) and associated guidelines. The procedures had also been approved by the appropriate ethics committee at Cardiff University.

#### 2.1.2 Surgical procedures

At the time of surgery the rats were approximately three months old and weighed between 250 g and 295 g. All rats were anaesthetized with isoflurane (4% induction, 2% thereafter). Next, each rat was placed in a stereotaxic frame (David Kopf Instruments, Tujunga, CA), with the incisor bar set at +5.0 mm to the horizontal plane. For analgesic purposes, Lidocaine was administered topically to the scalp (0.1 ml of 20 mg/ml solution; B. Braun, Melsungen, Germany) and meloxicam was given subcutaneously (0.06 ml of 5 mg/ml solution, Boehringer Ingelheim Ltd, Berkshire, UK). A craniotomy was then made directly above the target region and the dura cut to expose the cortex. Lesions of the anterior thalamic nuclei (ATN1) were made by injecting a cocktail consisting of 10 mg/ml N-methyl-D-aspartic acid (NMDA; Sigma, Poole, U.K.) and 10 mg/ml ibotenic acid (Tocris, Avonmouth, U.K.) dissolved in phosphate-buffered saline (pH 7.4) in two sites in each hemisphere using a 26 gauge, 1 μl Hamilton syringe (Hamilton, Bonaduz, Switzerland). The injection coordinates relative to bregma (in mm) were (1) AP -0.1, ML ± 0.8, DV -6.9; (2) AP -0.2, ML ± 1.5, DV -+6.3. The volumes injected were 0.16 μl and 0.20 μl respectively. The surgical control group (Sham1 controls) received identical treatment, except that no neurotoxin was infused into the brain.

### 2.2 Behavioural testing

Following recovery from surgery, the rats were first tested on T-maze alternation (Experiment 1 A). Next, the rats completed a latent inhibition task in operant boxes (see Nelson et al., in press), followed by a spontaneous object exploration task and then the present discrimination tasks (Experiments 1B-C, Table 1).

**Table 1:** Summary of the tasks used in Experiments 1B/C and 2 and the pattern of lesion-induced deficits associated with each task.

***Experiment 1B***		***Experiment 1C***		***Experiment 2***	
Strategy-shift (operant box)		Strategy-shift (water tank)		Response conflict (operant box)	
**Visual Discrimination**	*Unimpaired*	**Response Discrimination**	*Unimpaired*	**Conditional Discrimination**	*Unimpaired*
**Switch to Response**	*Impaired in first session*	**Response Reversal**	*Impaired*	**Congruent Trials** **(no conflict)**	*Unimpaired*
**Response Reversal**	*Unimpaired*	**Switch to Visual**	*Unimpaired*	**Incongruent Trials** **(conflict)**	*Impaired in first 10 s of stimulus presentation*
**Visual Reversal**	*Unimpaired*	**Visual Reversal**	*Unimpaired*		

#### 2.2.1 Experiment 1A – T-maze (Reinforced spatial alternation)

##### 2.2.1.1 Apparatus

Testing took place in a modifiable cross-maze. Each of the four arms was 70 cm long and 10 cm wide. The maze had wooden floors and clear Perspex walls (17 cm high). A barrier blocked the base of one arm to form a T-shaped maze. At the end of the two cross arms there was a circular food well in which sucrose pellets (45 mg, Noyes Purified Rodent Diet, Lancaster, NH) were placed during testing. The orientation of the T-maze and the start arm position remained constant throughout the experiment. An aluminium barrier could be positioned ∼25 cm from the end of the start arm to create a start area. The maze was elevated on a 94 cm high stand and was located in a rectangular room (280 cm × 280 cm × 210 cm) with salient visual cues.

#### 2.2.2 Pre-training

This began at least a week after surgery. On day one, the rats were introduced to the apparatus in pairs with sucrose pellets scattered on the floor. First, they were confined to the start arm for 5 min and then the choice arms for 5 min. On day two, the same procedure was followed but the rats were placed in the maze individually. On day three, the rats were again placed into the start or choice arms separately, but the sucrose pellets were only located within the food wells. On days four and five, single sucrose pellets were repeatedly placed in the food wells.

#### 2.2.3 Testing

All animals completed one session a day for four days, each session consisted of six trials. Each trial had two stages, a ‘sample run' followed by a ‘test run'. Before each trial, two sucrose pellets were placed in each food well and a metal barrier was placed at the junction point of the T-maze closing one choice arm. Another metal barrier in the start arm, created a start area. To begin the sample run, the rat was placed in the start area. The barrier was raised and the rat ran down the start arm to turn into the one open choice arm, which contained two sucrose pellets. For the test run, which followed approximately 10–15 s later, the rat was returned to the start area and the barriers by the start box and at the choice point were removed allowing free access to both choice arms, but now only the arm that was closed during the sample run was baited. Consequently, the rat only received reward if it alternated, i.e., chose the opposite arm to the sample run. The rat was deemed to have chosen an arm when it placed a hind foot within that arm; no retracing was allowed. The rat was allowed to eat the sucrose pellets and was then returned to the holding cage. If the rat selected the incorrect arm (i.e., the arm previously visited on the sample run), it was allowed to run to the end of the arm to find the empty food well, but then returned to its individual holding cage.

The rats were tested in groups of three or four, with each rat having one trial in turn. Each trial took approximately one minute, so that the inter-trial interval was ∼3 min for any given rat.

#### 2.2.4 Statistical analysis

The percent correct trials for each session was calculated for each rat. The performances of the two lesion groups were compared using a mixed ANOVA, with Session as the repeated measure and Lesion status as the between-subjects factor. Partial eta squared (η_p_²) is reported as an estimate of effect size. Mauchly's test was computed to test the assumption of sphericity of the within-subject variables, where this assumption is violated Greenhouse-Geisser corrected degrees of freedom are reported.

### 2.3 Experiment 1B – strategy shifts and reversals in an operant box

To test if anterior thalamic lesions affected the rats’ ability to acquire or switch between visual-based and response-based discriminations they were tested on an automated procedure conducted in an operant chamber [40], closely based on a task designed by Floresco et al. [31].

#### 2.3.1 Apparatus

Instrumental training was conducted in a set of eight operant boxes (Med Associates Inc., St Albans, VT), each measuring 240 mm high x 240 mm deep x 300 mm wide. The boxes were arranged in two rows of four against one wall of the test room. Each box had two aluminium walls, with a clear Perspex front, back, and ceiling. The grid floor comprised 19 parallel stainless-steel bars spaced 16 mm apart. Each operant box was housed in its own sound and light attenuating chamber. Each box had a single, central food magazine flanked by two response levers that could be retracted.

During training, sucrose pellet reinforcers (45 mg; P. J. Noyes, Lancaster, NH) were delivered into a recessed food magazine situated in the centre of the right-hand wall of the operant box. The magazine was fitted with a pair of infra-red detectors that recorded magazine entries. Flat response levers, which could be retracted, protruded to the left and right of the magazine. Above each lever was a stimulus light that was never illuminated during pre-training. Equipment control and data recording were via an IBM-compatible microcomputer equipped with MED-PC software (Med Associates Inc., St Albans, VT).

#### 2.3.2 Behavioural training

Pre-training: All animals received a single session of magazine training during which 20 sucrose pellets were delivered into the food magazine on a variable interval 60 s schedule (i.e., on average, one pellet per minute). Over the next two days the rats completed two sessions of continuous reinforcement (one lever on each day, counterbalanced across animals), during which one lever was inserted into the operant chamber and every lever press was reinforced. The animal was required to press the lever at least 50 times in 30 min before proceeding to the next stage; all animals met this criterion, so no additional training sessions were required.

The final stage of pre-training consisted of four or five sessions (one per day) for each animal. In each session, either the left or the right lever was presented on a given trial. The side on which the lever was presented was random for the first trial of a pair and the opposite lever was then presented on the subsequent trial. Trials commenced with illumination of the house light and the insertion of the lever. If the animal made a lever press response within 10 s of the lever being inserted a pellet was delivered, the lever retracted and, after 4 s, the house light was switched off. If the animal failed to respond within 10 s, the lever was retracted, the house light switched off and the trial counted as an omission. Each session consisted of 90 trials (45 left lever/ 45 right lever). Nineteen rats received four sessions. Eleven rats made more than five omissions in the 4th session and so they received an additional 5th training session.

#### 2.3.3 Discrimination training

**Fig. 1:**
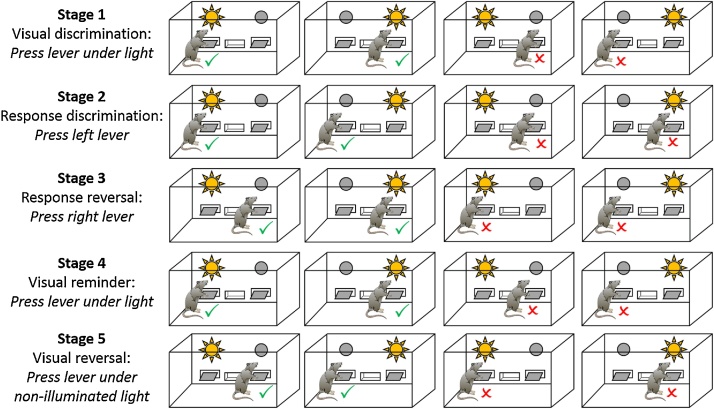
Strategy-shift task, Experiment 1B. Schematic showing the order of discrimination stages for the strategy-shift task conducted in operant boxes. A tick indicates a correct (reinforced) lever press for this particular animal. A cross indicates an incorrect lever press.

All animals learnt two discrimination strategies (visual and response stages), which required the use of different cues to earn food reinforcement (Fig. 1). They also completed sessions in which the correct response was reversed (visual reversal and response reversal stages). The initial visual discrimination was repeated later in the training to assess how the interposing discriminations influenced learning. Consequently, there were five sequential discriminations (Fig. 1). The order of the discrimination stages was the same for all animals.

Each session terminated when the animal had completed at least 60 trials and had reached a performance criterion of 10 consecutive correct responses, or after 120 trials. All animals received a minimum of two sessions on each discrimination stage. Each rat completed a stage when it made 10 consecutive correct responses in each of two consecutive sessions, with the additional requirement that they made fewer than 20% errors on the final session. These strict criteria helped to ensure that the animals had thoroughly mastered each discrimination before required to switch to a new strategy. As there was no subsequent switch in strategy following the final ‘Visual Reversal’ discrimination only the first criterion was applied, consequently, testing finished when an animal made 10 consecutive correct responses.

#### 2.3.4 Visual discrimination

All animals completed the Visual Discrimination first. For this condition, one of the stimulus lights (left or right) was illuminated at the start of each trial. Three seconds later both levers were inserted into the operant chamber and the house light illuminated. A response on the lever below the illuminated stimulus light (correct response) resulted in a single pellet being delivered in the magazine, the extinguishing of the stimulus light and both levers retracted. After 4 s the house light was extinguished, signalling the start of the 20 s inter-trial interval (ITI). Following an incorrect response (i.e., a response on the lever under the non-illuminated stimulus light) the chamber immediately reverted to the ITI state. If an animal failed to respond within 10 s of the trial starting, the chamber also reverted to the ITI state. The position of the correct lever (left or right) was random for the first trial of a pair, while the correct lever in the next trial was always on the opposite side. Thus, for odd numbered trials there was an equal probability of either the left or right lever being rewarded, whilst on the subsequent trial the opposite lever was always rewarded.

#### 2.3.5 Response discrimination

The structure of the Response Discrimination trials was essentially the same as those in the Visual Discrimination stage. However, during this stage, either the left or right lever was designated as the ‘correct’ lever (counterbalanced across animals); only responses on this lever were reinforced regardless of the position of the illuminated stimulus light (Fig. 1). The stimulus light was still presented above one of the levers so that, over the course of the session, for half of the trials the light was illuminated above the correct lever (‘Congruent trials’) and for remaining trials the light was above the incorrect lever (‘Incongruent trials’).

#### 2.3.6 Response reversal training

After reaching criterion on the Response Discrimination, the lever contingencies were reversed such that the previously incorrect lever was now reinforced and *vice versa* (‘Response Reversal’). All other conditions remained the same as in the previous stages.

#### 2.3.7 Visual reversal training

After reaching the performance criteria on the Response Reversal, training was paused for some animals to allow all animals to complete this stage. The training sessions then reverted back to the initial visual discrimination contingency (‘Visual Reminder’). Once a rat again reached performance criteria, the visual discrimination contingency reversed such that now the animal had to press the lever under the non-illuminated stimulus light to receive reward pellets (‘Visual Reversal’). Now, the stimulus light signalled the incorrect lever.

#### 2.3.8 Statistical analysis

The mean number of errors required for the two groups to reach criterion on the first ‘Visual Discrimination’ were initially compared in a two-sample *t*-test in order to ensure that the groups were starting from the same baseline and any differences seen following subsequent strategy switches were not due to initial learning differences.

The mean number of trials to criterion and errors to criterion for each stage were compared between the groups using mixed ANOVAs with the within-subject factor ‘Stage’ and the between-subject factor ‘Lesion’. To examine more closely the effects of a strategy switch, the first session of each stage, i.e., when a new strategy was introduced, was divided into blocks of 10 trials. The first six blocks (i.e., 60 trials) were analysed using a mixed ANOVA with the within-subjects factors of ‘Stage’ and ‘Block’ (1–6) and between-subjects factor of ‘Lesion Group’. Sixty trials were selected as this is the minimum number that all rats completed. Partial eta squared (η_p_²) is reported as an estimate of effect size. To test the assumption of sphericity of the within-subject variables Mauchly's test was calculated; where significant the Greenhouse-Geisser corrected degrees of freedom are reported.

In addition, for the Response Discrimination and Visual Reminder stages (i.e., stages where a strategy shift was required, as opposed to a simple reversal), trials were classified according to whether or not the correct lever was the same as it would have been for the previous discrimination (i.e., Congruent trials) or different (i.e., Incongruent trials). For example, during the Response Discrimination stage the light was illuminated above the correct lever (i.e., a Congruent trial) on half of the trials, meaning that the discrimination could be solved using the previously learnt strategy (i.e., press the lever with the light above it). Conversely, in order to select the correct lever on Incongruent trials (i.e., when the light was illuminated above the incorrect lever) the animal had to inhibit the previously learnt strategy. Therefore, errors made on Incongruent trials were categorised as ‘Perseverative’. Perseverative errors rates were analysed for the first six blocks (i.e., 60 trials) of the first Response Discrimination session and, in a separate ANOVA, for the first six blocks of the first Visual Reminder session.

### 2.4 Experiment 1C – strategy shifts and reversals in a water tank

**Fig. 2:**
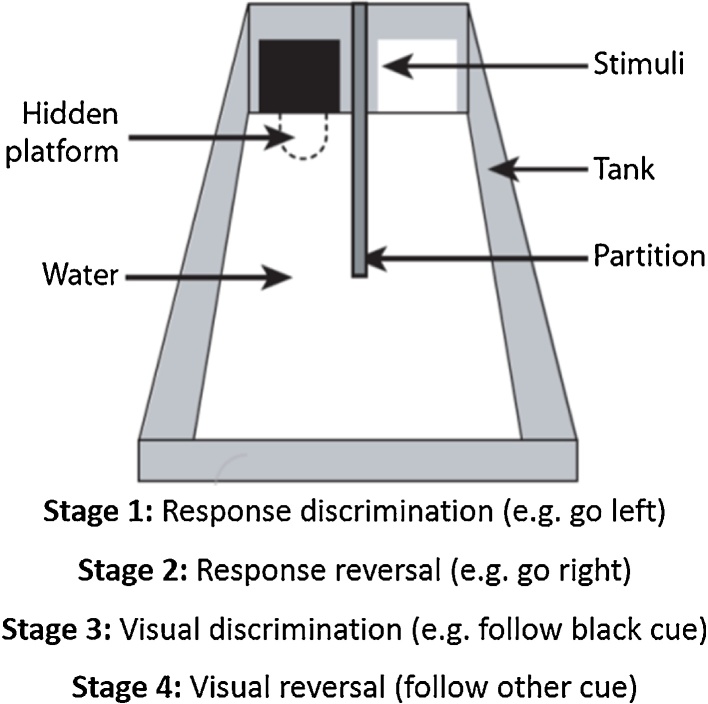
Strategy shifts and reversals in a water tank, Experiment 1C. Schematic showing the testing apparatus and order of the discriminations.

The same cohort of rats received a series of two-choice discriminations in a water tank (Fig. 2). The escape location was first specified by an egocentric discrimination (left vs. right) and later by a visual discrimination (black vs. white). For both discriminations the contingencies were also reversed (Fig. 2).

#### 2.4.1 Apparatus

Testing took place in a grey opaque, acrylic tank that sat within a circular water maze (2 m diameter). The test tank was 100 cm long, 62 cm wide, and 62 cm deep with a partition wall (measuring 62 cm high and 46 cm long) projecting at right angles from the middle of the end wall (the stimulus wall), creating two goal areas (Fig. 2). The tank was filled to a depth of 32 cm with water made opaque by adding a nontoxic emulsion (opacifier E308, Chesham Chemicals, Harrow, UK). The water temperature remained between 23 and 26 °C. For every trial a circular, submerged escape platform (2 cm thick, 12 cm diameter, clear acrylic) was located beneath one of the stimulus walls (Fig. 2). This transparent escape platform was located 2 cm below the water surface and was not visible. The water maze was surrounded by a white circular curtain throughout training to reduce the use of extra-maze cues.

The two stimuli used for the visual discrimination were one black and one white cue card, both laminated to keep them waterproof. These stimuli measured 210 × 210 mm and were attached to the centre of each stimulus wall (Fig. 2) with their bottom edges submerged just below the water.

#### 2.4.2 Behavioural training

Pre-training: All rats were first pre-trained to find the hidden platform in the absence of any test stimuli. During pre-training, each rat received 12 trials per session over two sessions to locate the two possible platform positions. Pre-training started by placing the rat on the escape platform in one of the goal (escape) areas. For each pre-training trial the rat was placed progressively further away from the platform and allowed to follow the experimenters hand to guide it to the platform. This procedure occurred six times for the left goal area and six times for the right goal area in each session in a pseudorandom order. By the end of the second pre-training session, all rats could be placed facing the start wall, where they would turn and swim to search for the escape platform in the two possible positions.

#### 2.4.3 Discrimination training

Throughout all training, the escape platform was located directly under the midline of the reinforced stimulus (S+; see Fig. 2). For the visual discrimination, the S + appeared equally often in the right and left goal areas in a pseudorandom order, with the constraint that an S + could not appear in the same goal location on more than two consecutive trials. If a rat swam to the S + it was allowed to sit on the platform for 10 s before being removed and placed in a dry box. When a rat swam to the incorrect goal location (S–, no platform), it was allowed to continue swimming to return around the partition wall to reach the platform in the other goal area, that is, the rat was allowed to self-correct. An incorrect trial was recorded if a rat’s snout came within 15 cm of the S–. The rats were tested sequentially in groups of three or four, resulting in an interval of 4–5 min between trials for an individual rat. There were twelve trials per session. Each rat completed a stage when it reached a criterion of at least 80% correct trials in two consecutive sessions.

#### 2.4.4 Stage 1 - response discrimination

For any given rat, for every trial in this stage the escape platform was always in the same goal area. Reinforcement of the left or right goal area was counterbalanced across the surgical groups. The black and white cues were present for every trial and their placement in the left or right goal area was in a pseudorandom order so they were irrelevant to solving the task.

#### 2.4.5 Stage 2 - response reversal

For this stage there was a simple contingency reversal; those rats that were previously rewarded for going left were now rewarded for going right and *vice versa*. The black and white cues remained present but irrelevant for solving the task.

#### 2.4.6 Stage 3 - visual discrimination

At this stage the black and white cues became relevant to solving the discrimination. Now, the location of the escape platform was indicated by either the black or white cue. The colour assigned as S + for each rat was counterbalanced across the surgical groups and with respect to their previous contingencies in the response stages.

#### 2.4.7 Stage 4 - visual discrimination reversal

In the final stage the visual contingency was reversed. Rats previously trained to find the escape platform by swimming to the black cue now swam to the white cue and *vice versa*.

#### 2.4.8 Statistical analysis

The mean number of errors required to reach criterion for each stage were compared between the groups using a mixed ANOVA with the within-subject factor ‘Stage’ and the between-subject factor ‘Lesion’. To examine if the particular types of strategy shift were learned differently by the lesion groups a separate mixed ANOVA compared the mean number of errors to criterion with two within-subject factors of ‘Stimulus Class’ (visual or response) and ‘Switch Type’ (strategy switch or reversal), and the between-subject factor of ‘Lesion’. Partial eta squared (η_p_²) is reported as an estimate of effect size. To test the assumption of sphericity of the within-subject variables Mauchly's test was calculated; where significant the Greenhouse-Geisser corrected degrees of freedom are reported.

### 2.5 Histology and lesion analysis

On completion of behavioural testing, the rats received a lethal overdose of sodium pentobarbital (60 mg/kg, IP, Euthatal, Rhone Merieux) and were transcardially perfused with 0.1 M phosphate-buffered saline followed by 10% formal saline. The brains were removed and post-fixed overnight in formal saline, then incubated in 25% sucrose at room temperature overnight on a stirrer. The brains were cut in the coronal plane into 40 μm sections using a freezing microtome (Leica). A 1 in 4 series of sections was mounted directly onto gelatine-subbed glass slides and then stained with cresyl violet.

### 2.6 Results

#### 2.6.1 Histology

**Fig. 3:**
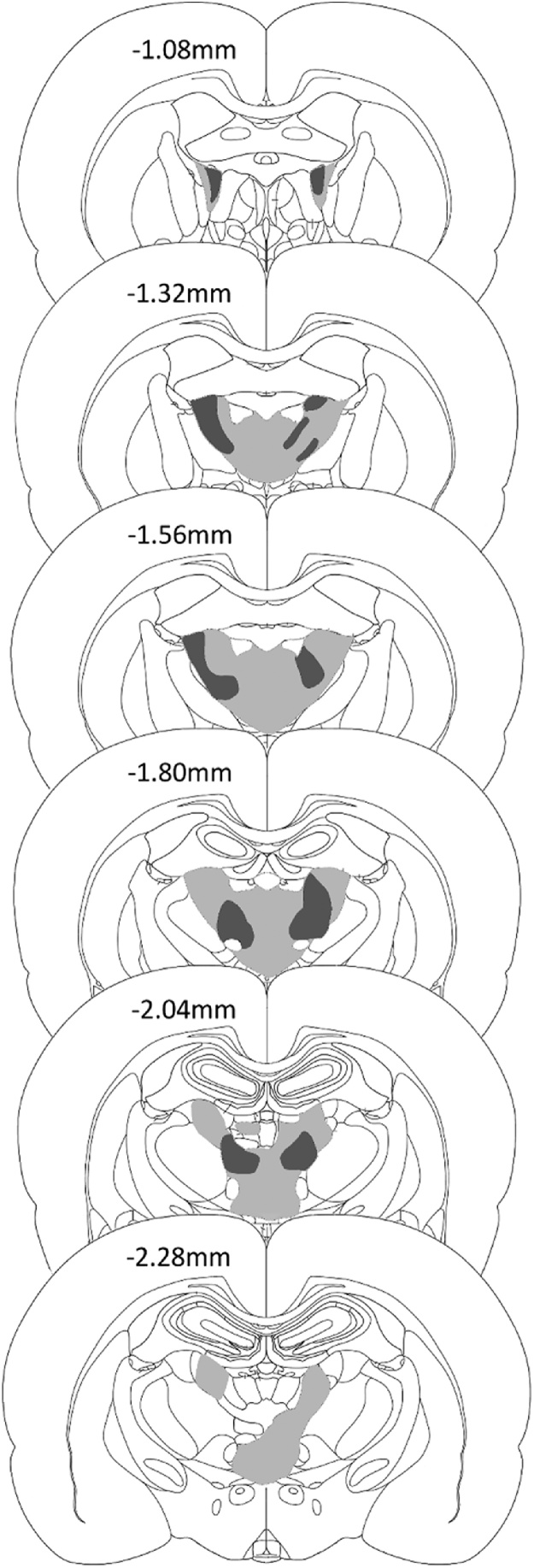
Location and extent of anterior thalamic nuclei lesions of group ATN1. The coronal reconstructions show the cases with the minimal (dark grey) and maximal (light grey) extent of anterior thalamic nuclei tissue loss. The numbers indicate the distance (in millimetres) from bregma adapted from Paxinos & Watson [60].

The three ATN1 cases with the smallest anterior thalamic lesions also had evident fornix disruption. Accordingly, these three cases were excluded. In addition, two control animals with unexpected damage to the fornix were also excluded, leaving groups sizes of ATN1 = 13, Sham1 Controls = 12 (Fig. 3).

Of the thirteen ATN1 cases, there was complete, bilateral loss of the anterior thalamic nuclei in five cases. These larger lesions consistently resulted in additional cell loss in the rostral paraventricular nucleus, rostral nucleus reuniens and parts of the parataenial nucleus (Fig. 3). In two further cases, there was complete loss of the anterior thalamic nuclei in one hemisphere, with limited sparing of subregions within the anteroventral nucleus in the other hemisphere. There was associated reuniens cell loss in one of these cases. In a further four cases, the lesions were more restricted, leading to a loss of 60–70% of the anterior thalamic nuclei, but with little or no additional damage. Finally, two cases had bilateral cell loss centred at the junctions of the anteromedial and anteroventral thalamic nuclei, with only partial asymmetric damage in the remaining parts of the thalamic group (Fig. 3). In two of the thirteen cases, there was restricted cell loss at the rostral limit of the medial dorsal thalamic nucleus, while the rostral border of the lateral dorsal nucleus was affected in four animals. Finally, in six cases there was an extremely limited patch of cells loss at the very rostral limit of the medial blade of the dentate gyrus in the septal hippocampus. This hippocampal damage was unilateral in five of the six cases.

#### 2.6.2 Experiment 1A - T-maze alternation

**Fig. 4:**
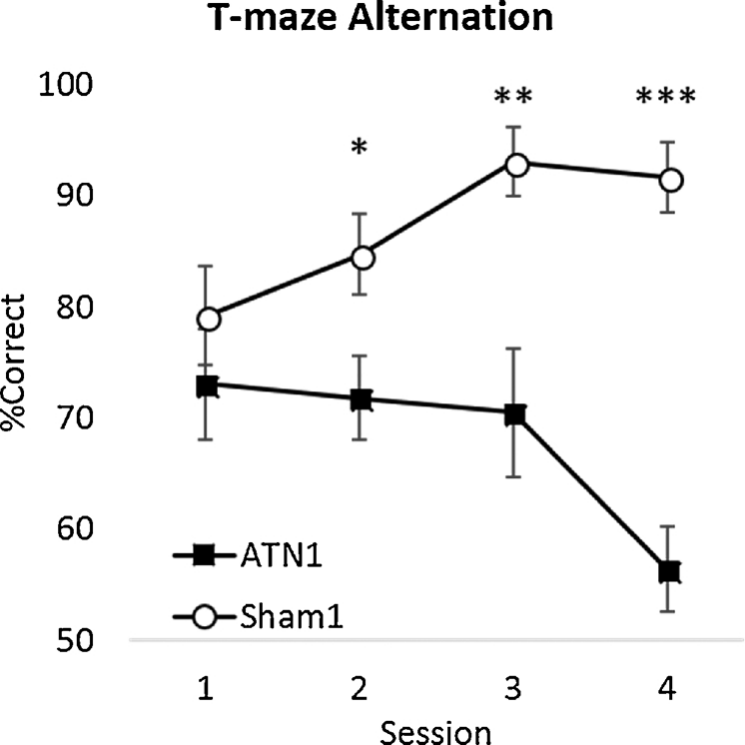
T-maze Alternation, Experiment 1 A. The mean percentage of correct responses across four sessions of acquisition by rats with anterior thalamic lesions (ATN1) and surgical controls (Sham1). Fifty percent represent chance (i.e., the likelihood of choosing either arm in the T-maze). Error bars ± standard error of the mean. * p < 0.05, **p < 0.01. ***p < 0.001.

The ATN1 group were significantly impaired relative to their Sham Controls (F_1,22_ = 33.2, p < 0.001, η_p_² = 0.60). While there was no effect of session (F_3,66_ = 1.52, p = 0.22, η_p_² = 0.07) there was a significant interaction (F_3,66_ = 4.22, p = 0.009, η_p_² = 0.16) as the rats with ATN lesions performed significantly worse than controls in the later sessions (Fig. 4).

#### 2.6.3 Experiment 1B – discrimination shifts and reversals in an operant box

**Fig. 5:**
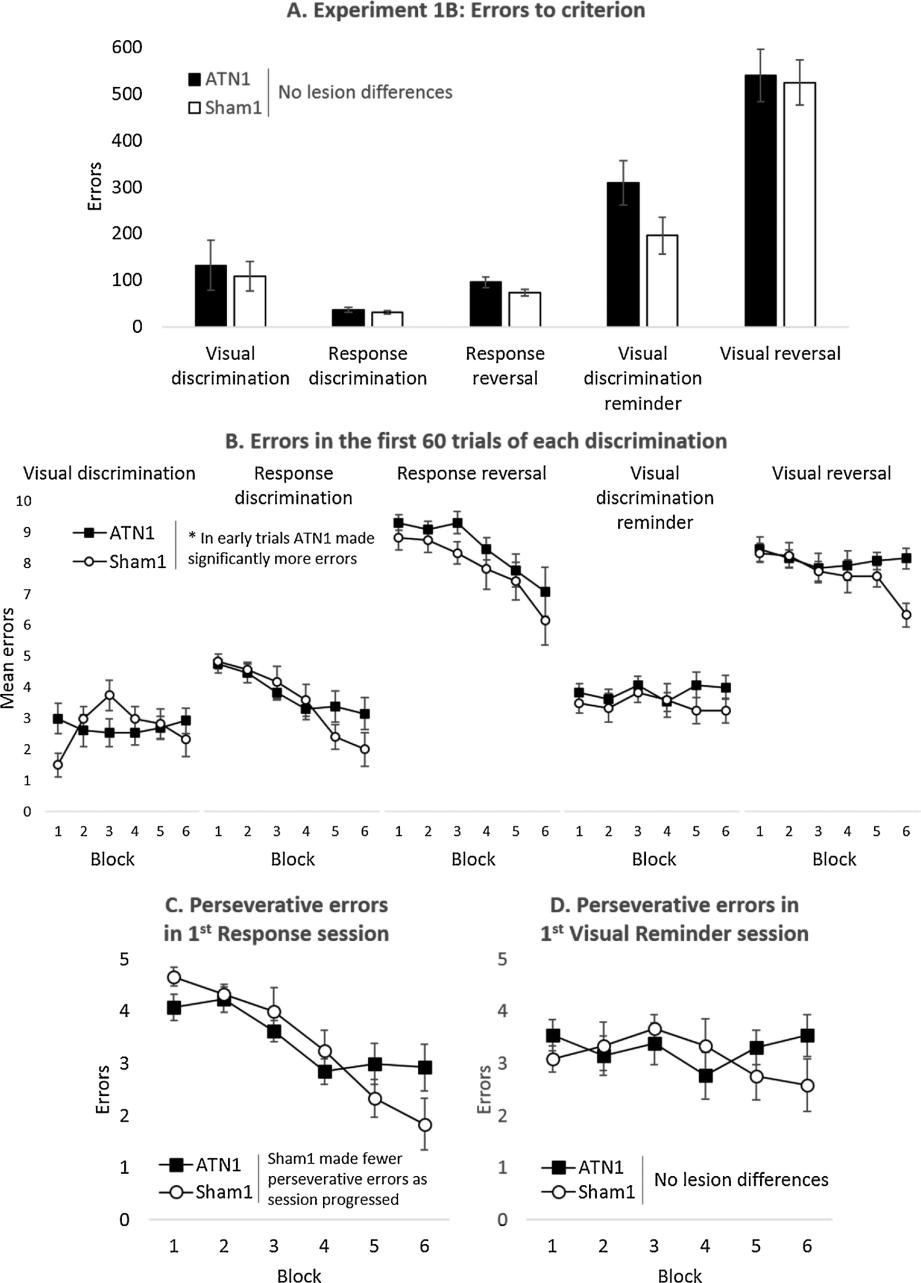
Strategy-shift task, Experiment 1B. (A) Mean errors to reach criterion for each discrimination stage; there were no differences between the lesion groups on this measure. (B) Mean errors made during the first six blocks (60 trials) of each discrimination stage; overall, the ATN1 group made more errors than the Sham1 group in these initial trials of each stage. (C) Perseverative errors during the first six blocks of the Response discrimination; Sham1 group made fewer perseverative errors as the session progressed. (D) Perseverative errors during the first six blocks of the Visual Reminder Session; there were no differences between the lesion groups on this measure. ATN1, rats with anterior thalamic lesions; Sham1, surgical controls. Error bars ± standard error of the mean.

The mean number of trials required to complete the first discrimination, when the animals learnt to press the lever under the illuminated light to gain a reward was 472 ± 167 (ATN1) and 384 ± 92 (Sham1 Controls). There was no statistical difference between the surgical groups (t_23_ = 0.45, p = 0.66). Likewise, there was no statistical difference in the number of errors made to reach criterion (ATN1 132 ± 54; Controls 108 ± 32; t_23_ = 0.36, p = 0.72; Fig. 5A). These null results indicate that the ATN1 group could learn this type of discrimination at the same rate as their controls, so permitting unbiased assessments when subsequently switching contingencies.

To assess if the ATN1 lesions affected learning rates across all five discriminations, the number of errors to reach criterion on each discrimination stage was examined. There were no lesion differences in the number of errors to reach criterion on each discrimination stage (F_1,23_ = 1.28, p = 0.27, η_p_² = 0.05; Fig. 5A) nor was there a Lesion x Stage interaction (F_2.2,50.5_ = 0.81, p = 0.52, η_p_² = 0.03). A significant Mauchly’s test (p ≤ 0.001) indicated that the assumption of sphericity was violated and so Greenhouse-Geisser corrected degrees of freedom are presented for the within-subjects contrast. Likewise, there were no group differences in the number of trials or the number of sessions required to reach criterion (data not shown).

The first session of each discrimination stage should be the most sensitive to the challenge of learning a new reinforcement contingency, especially when influenced by the previous discrimination. For this reason, the numbers of errors made in the first session across all discriminations were examined (Fig. 5B). Overall, the ATN1 group made significantly more errors in the first six blocks (i.e., 60 trials) of the first session of each discrimination (F_1,23_ = 7.23, p = 0.013, η_p_² = 0.24) but this lesion effect did not differ between the discriminations (F_4,92_ = 0.28, p = 0.89, η_p_² = 0.01). There was a significant interaction between Block x Lesion group (F_5,115_ = 3.78, p = 0.003, η_p_² = 0.14), reflecting how the error rates of the ATN1 group typically did not fall as each first session progressed. This effect appeared to be nonspecific as the three-way interaction was not significant (F_20,460_ = 1.03, p = 0.43, η_p_² = 0.04). Thus, in this type of operant task, the ATN1 lesions increased error rates when a new strategy is reinforced but the animals recover from this initial deficit.

For the Response Discrimination and Visual Reminder stages, in which a strategy shift was required (rather than a reversal), trials that were incorrect on the current discrimination but would have been correct on the previous discrimination were categorised as ‘Perseverative errors’ (see Methods section). There was no overall difference between the ATN1 group and their Sham1 Controls in the number of perseverative errors made during the first six blocks of the first Response Session (F_1,23_ = 0.018, p = 0.90, η_p_² = 0.001; Fig. 5C). There was, however, a Lesion by Block interaction (F_5,115_ = 2.94, p = 0.015, η_p_² = 0.11) indicating that the distribution of perseverative errors across the first session differed by lesion group, with the control group making fewer errors towards the end of the session. The same analysis for the first session of the Visual Reminder stage found no lesion effect (F_1,23_ = 0.13, p = 0.72, η_p_² = 0.006; Fig. 5D) or Lesion x Block interaction (F_5,115_ = 1.76, p = 0.13, η_p_² = 0.07) indicating that there were no differences in the way the groups re-acquired the Visual discrimination rule over the first 60 trials of Session 1.

#### 2.6.4 Experiment 1C – strategy shifts and reversals in a water tank

**Fig. 6:**
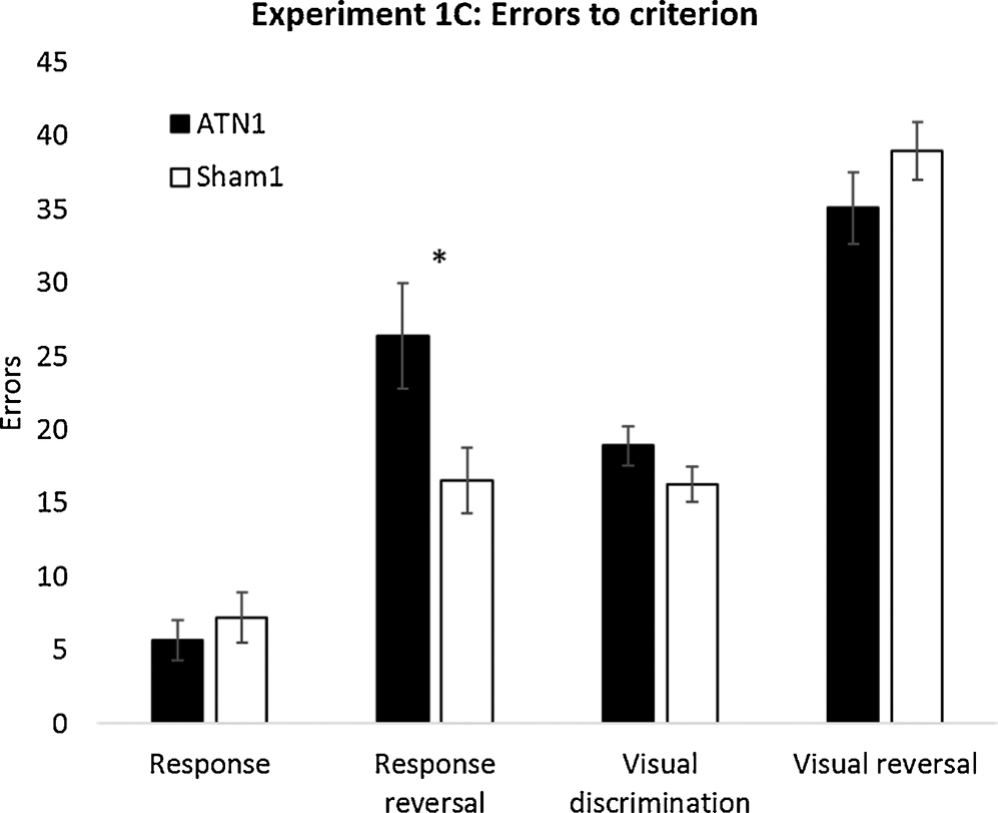
Strategy shifts and reversals in a water tank, Experiment 1C. Mean errors to criterion for the four discriminations (including reversals) conducted in the water tank. ATN1, rats with anterior thalamic lesions; Sham1, surgical controls. Error bars ± standard error of the mean. * p < 0.05.

One animal in the control group could not master the swimming pre-training and so was dropped from the study, leaving ATN1 = 13, Sham1 = 11. Inspection of Fig. 6 suggests that the ATN lesions had a selective, disruptive effect on the response reversal condition. This impression was supported by subsequent statistical analyses.

Initially, the number of errors to reach criterion on each discrimination stage was examined. There was no overall lesion difference (F_1,22_ = 1.49, p = 0.24, η_p_² = 0.06; Fig. 6), however, the Lesion x Stage interaction was significant [F_2.3,50_ = 3.33, p = 0.038, η_p_² = 0.13 (Greenhouse-Geisser corrected degrees of freedom)] indicating that the ATN lesions affected the discriminations differently. To identify which discrimination was affected an ANOVA with the factors ‘Stimulus Class’ (Visual or Response) and ‘Switch Type’ (Acquisition or Reversal) was calculated. This analysis revealed that both groups of rats found reversals more difficult than the initial acquisition of a rule (F_1,23_ = 115, p < 0.001, η_p_² = 0.84). Additionally, both groups took more trials to learn the visual discriminations relative to the response (egocentric) discriminations (F_1,22_ = 71.7, p < 0.001, η_p_² = 0.77; Fig. 6). There was no interaction between Discrimination type and Lesion (F_1,22_ = 2.32, p = 0.14, η_p_² = 0.1) or between Reversal and Lesion (F_1,22_ < 1, η_p_² = 0.02), however, the three-way interaction was significant (F_1,22_ = 6.45, p = 0.019, η_p_² = 0.23), i.e., the ATN lesions selectively impaired the response reversal. Follow-up simple main effects analysis confirmed this; there was a significant difference between the lesion groups on the Response Reversal (F_1,22_ = 4.58, p = 0.044) while there was no difference between the lesion groups on each of the other discriminations (Response: F_1,22_ < 1; Visual Discrimination: F_1,22_ = 1.91, p = 0.18; Visual Reversal: F_1,22_ = 1.39, p = 0.25).

## 3 Experiment 2 – response choice during stimulus conflict

Experiment 2 assessed the impact of ATN damage on a rat analogue of the Stroop task that measures behavioural flexibility in response to conflict as well as the use of higher order rules to guide instrumental behaviour [34,41]. Rats concurrently learn two conditional discriminations, one visual and one auditory, in two distinct contexts. At test, animals receive compound audiovisual stimuli either composed of those stimulus elements that had elicited the same response (‘congruent’ trials) or different responses (‘incongruent’ trials) during training. Responses during incongruent stimulus compounds are defined as correct or incorrect according to whether they are appropriate to the test context.

### 3.1 Materials and methods

#### 3.1.1 Animals

The experiment involved 20 adult male Lister Hooded rats (Charles River, UK). The rats weighed 270–320 g at the beginning of the experiment. Details of housing and husbandry are the same as described for Experiment 1. Rats were randomly assigned to one of two groups prior to surgery; anterior thalamic nuclei (ATN2, n = 10) or surgical controls (Sham2, n = 10). All procedures were in accordance with the UK Animals (Scientific Procedures) Act, 1986 and EU directive (2010/63/EU), as well as being approved by local ethical committees at Cardiff University

#### 3.1.2 Surgical procedures

For 17 of the 20 rats the surgery was performed under an isoflurane-oxygen mixture (1.5–2.5% isoflurane) with a reduced dose of sodium pentobarbital (14 mg/kg, i.p) when the surgery was nearing completion. For three rats, the surgeries involved just sodium pentobarbital anaesthesia (60 mg/kg i.p., Sigma-Aldrich Company Ltd, Dorset, UK). All other aspects of surgery were the same as described for Experiment 1 except that the lesions were made by injecting 0.12 M N-methyl-D-aspartic acid (NMDA; Sigma Chemicals UK) dissolved in sterile phosphate buffer (pH 7.4). The injection site co-ordinates were: medial injections, AP -0.1, ML ± 0.8, DV -6.8; lateral injections, AP -0.4, ML ± 1.5, DV -6.2. The injected volume of the medial injections was 0.20 μl, while the more lateral injections were 0.18 μl of 0.12 M NMDA. The surgical controls (Sham2) were treated identically except that no neurotoxin was injected.

After removal of the Hamilton syringe, the incision was cleaned and sutured. A topical antibiotic powder (Aureomycin, Fort Dodge, Animal Health, Southampton, UK) was applied. The rats received glucose-saline (5 ml s.c.) for fluid replacement and were then placed in a recovery chamber until they regained consciousness. Rats were given the analgesic Metacam (0.06 ml s.c.; 5 mg/ml meloxicam; Boehringer Ingelheim Vetmedica, Germany). A respiratory stimulant millophylline (0.1 ml s.c., Arnolds Veterinary Products, Shropshire, UK), an antibiotic in their water (Baytril 2.5%; Bayer Ltd, Animal Health Division, Ireland), and a low dose of diazepam (0.07 ml s.c., 5 mg/ml; CP Pharmaceuticals Ltd, UK) were administered to facilitate post-operative recovery. All animals were monitored carefully until they had fully recovered.

#### 3.1.3 Apparatus

**Fig. 7:**
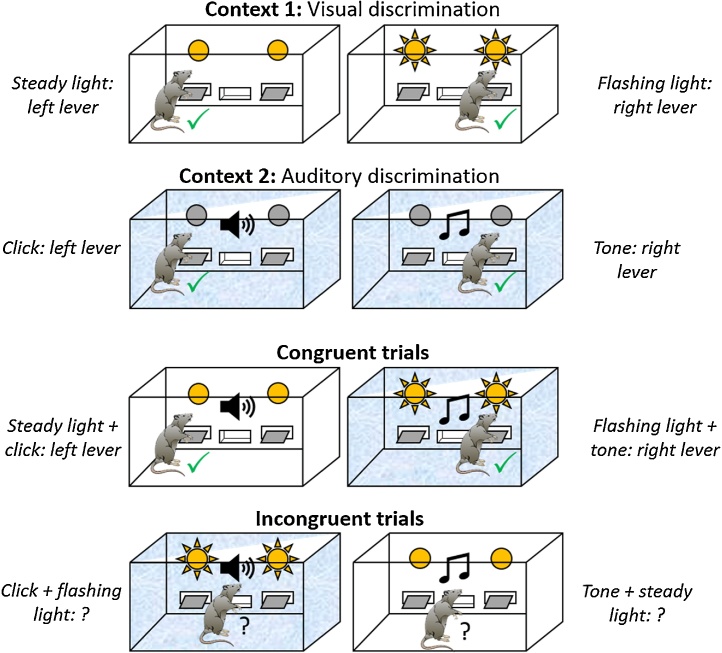
Response choice during stimulus conflict, Experiment 2. Schematic of the experimental design. Animals acquired two conditional discriminations (one auditory and one visual) in two distinct contexts with different rewards (food pellets and sucrose solution). During extinction tests, animals received audiovisual compounds of these training stimuli. These compounds comprised either elements that had elicited the same response (‘Congruent trials’) or different responses (‘Incongruent trials) during training. A tick indicates a correct (reinforced) lever press for this particular animal during initial conditional discrimination training. For the congruent test trials there is a ‘correct’ response (tick) but for the incongruent test trials the animals have to use contextual information to disambiguate the conflicting cues.

Eight operant chambers (30 cm wide x 24 cm deep x 21 cm high; Med Associates, George, VT) were used of two distinctive types. Each chamber had three aluminium walls, with a Perspex door serving as the fourth wall. In four ‘white’ chambers, the walls and ceilings were lined with white paper with a single 5 cm black stripe, fixed behind transparent Perspex (Context 1). The other four chambers were ‘plain’ aluminium (Context 2; Fig. 7). Each chamber floor consisted of 19 stainless-steel rods (3.8 mm in diameter, spaced 1.6 cm apart). In four chambers the sawdust beneath the floor was mixed with cumin powder, in the other four it was mixed with paprika powder. Each chamber was illuminated by a 3 W house-light located at the top centre of the left wall. Food pellets (45 mg; Noyes, Lancaster, NH) could be delivered into a recessed magazine located in the centre of the right chamber wall. Fifteen percent sucrose solution could be delivered via a dipper into the same magazine. Two flat-panel retractable levers were located to the left and right of the magazine. Auditory stimuli consisted of a 2kHZ tone and 10 Hz train of clicks, both delivered through ceiling speakers. Visual stimuli consisted of either two ‘flashing’ (0.1 s on, 0.1 s off) panel lights (each 2 cm diameter, located above the retractable levers) or two ‘steady’ panel lights plus illumination of the magazine light.

#### 3.1.4 Behavioural testing

##### 3.1.4.1 Lever press training

Rats received four training sessions, during which a rat would lever press for a single food pellet or 0.1 ml of the sucrose solution on a random interval schedule (RI15) such that once in every 15 s, on average, a reward became available following a lever press.

##### 3.1.4.2 Conditional discrimination training

Next, rats learnt two concurrent conditional discriminations for 18 days. There were two sessions a day, one in each of the two contexts (e.g., white/cumin and plain/paprika). One session was conducted in the morning and the other in the afternoon (minimum of four hours between each session). Correct responses were rewarded with food pellets in one context and sucrose solution in the other.

In one context (e.g., white chamber) rats were presented with visual cues (flashing or steady lights). During one visual stimulus (e.g., steady lights), only responding on the left lever was reinforced; during the other visual stimulus (e.g., flashing lights), only responding on the right lever was reinforced. In the second context (e.g., plain chamber), auditory stimuli were used (click or tone). For one auditory stimulus (e.g., click) only responding on the left lever was reinforced, while for the other auditory stimulus (e.g., tone) only responding on the right lever was reinforced (Fig. 7).

The contexts, stimuli, responses and rewards were counterbalanced across animals as far as possible, ensuring that each lesion group experienced both of the discriminations (auditory and visual) in both contexts (white or plain) with both rewards (sucrose and pellets).

Each session consisted of 24 trials. In one context, a session comprised 12 tones and 12 clicks. In the second context, a session comprised 12 steady and 12 flashing lights. There was a mean inter-stimulus interval of 60 s (range 30 s to 90 s). Both levers were present during each stimulus presentation and retracted during the inter-stimulus interval. Each stimulus presentation lasted 60 s. During the first 10 s of each trial, reinforcement was unavailable so that discrimination performance was uncontaminated by reinforcement. During the remaining 50 s, reinforcement was available on the RI15 schedule of reinforcement (see above).

##### 3.1.4.3 Extinction sessions

All rats next received four extinction sessions: two in each of the two training contexts. The animals first received two days of extinction testing (one in each context) and then, after two days of reminder training on the original conditional discriminations, two more extinction sessions. The test order (Context 1 versus Context 2) was counterbalanced across animals. Extinction testing consisted of presenting either individual training stimuli (‘single element’) or audiovisual compounds of the training stimuli (‘congruent’ and ‘incongruent’, see Fig. 7). Rats received 12 extinction trials in total per session (four single element, four congruent, and four incongruent) and for each trial type there were two possible stimuli or stimulus compounds. Trial order was block randomized, with each stimulus or compound being presented once in each block of six trials. Both levers were available but responding was not reinforced. Stimulus duration was 60 s and there was a mean inter-stimulus interval of 60 s.

Congruent stimulus compounds consisted of visual and audio elements that had been trained to elicit the same lever response in both contexts. For example, if both click and steady light had signalled a rewarded left lever press, when presented together both stimuli should elicit the same lever response irrespective of context. In contrast, incongruent stimulus compounds comprised individual elements that after training elicited different responses. For example, within the incongruent compound ‘flashing light + click’, the flashing light elicited a right lever press in Context 1 but the click elicited a left lever press in Context 2; for further details see [36].

#### 3.1.5 Statistical analysis

When performance on the conditional discrimination training was considered, response rates were calculated using only the first 10 s of stimulus presentation (during which no reinforcement was available) and expressed as a rate of lever presses per minute. These data were analysed by ANOVA with a between-subjects factor of Group (ATN2 or Sham2) and within-subject factors of Lever (correct and incorrect) and Block (9 blocks of two sessions). For the extinction test sessions, rates were calculated for the entire stimulus presentation (60 s) and again expressed as a rate of lever presses per minute on both the correct and the incorrect levers. ANOVAs with a between-subjects factor of Group (ATN2 and Sham2) and a with-subject factor of Lever (correct and incorrect) were carried out separately on each trial type (single-element, congruent and incongruent compounds). For incongruent test trials, responding according to the element that had previously been trained in that test context (i.e. context-appropriate) was deemed to be a correct response, while responding according to the element that had previously been trained in the alternative context (i.e. context inappropriate) was deemed an incorrect response. As previous work has shown that anterior cingulate lesion effects on incongruent trials are found during initial stimulus presentation [34], the responding during the first 10 s of stimulus presentation (incongruent trials) was also analysed. Partial eta squared (η_p_²) is reported as an estimate of effect size. To test the assumption of sphericity Mauchly's test was calculated; where significant the Greenhouse-Geisser corrected degrees of freedom are reported.

#### 3.1.6 Histology and lesion analysis

Histological procedures and lesion analysis proceeded as described for Experiment 1.

### 3.2 Results

#### 3.2.1 Histology

**Fig. 8:**
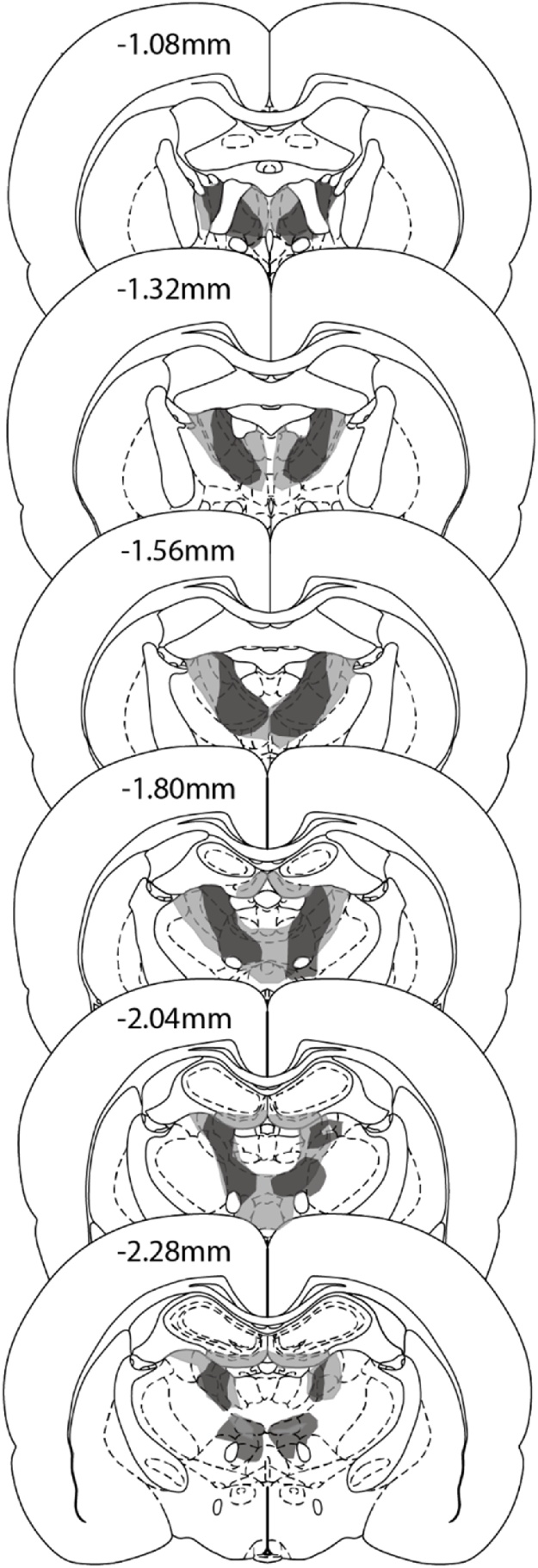
Location and extent of anterior thalamic nuclei lesions of group ATN2. The coronal reconstructions show the cases with the minimal (dark grey) and maximal (light grey) extent of anterior thalamic nuclei tissue loss. The numbers in indicate the distance (in millimetres) from bregma adapted from Paxinos & Watson [60]. (For interpretation of the references to colour in this figure legend, the reader is referred to the web version of this article).

Of the ten ATN2 rats, two had excessive, unintended cell loss within the medial dentate gyrus of the septal hippocampus, and were therefore removed. In the eight remaining cases, the anterior thalamic nuclei lesions were either essentially complete (n = 4) or a small island of cells within the anterior ventral nucleus was visible in just one hemisphere (Fig. 8). The lesions typically extended into adjacent midline nuclei such as the paraventricular nucleus (n = 4) and parataenial nucleus (n = 4, three of which had only unilateral cell loss). The lesions also extended ventrally to reach the very rostral part of the reticular nucleus and the ventral anterior nucleus (both, n = 3). The rostral nucleus reuniens was involved in seven cases. More caudal nuclei such as the medial dorsal thalamic nucleus (unilateral, three cases) and the lateral dorsal nucleus (three cases, two of which unilateral) were occasionally involved at their rostral limit. Cell loss within the hippocampus was seen in only three cases where it was typically restricted to the medial blade of the dentate gyrus in the most rostral part of the septal hippocampus. A more common feature was that the third and lateral ventricles appeared enlarged.

#### 3.2.2 Experiment 2 – rodent analogue of the ‘Stroop’ task

##### 3.2.2.1 Acquisition of conditional discriminations

**Fig. 9:**
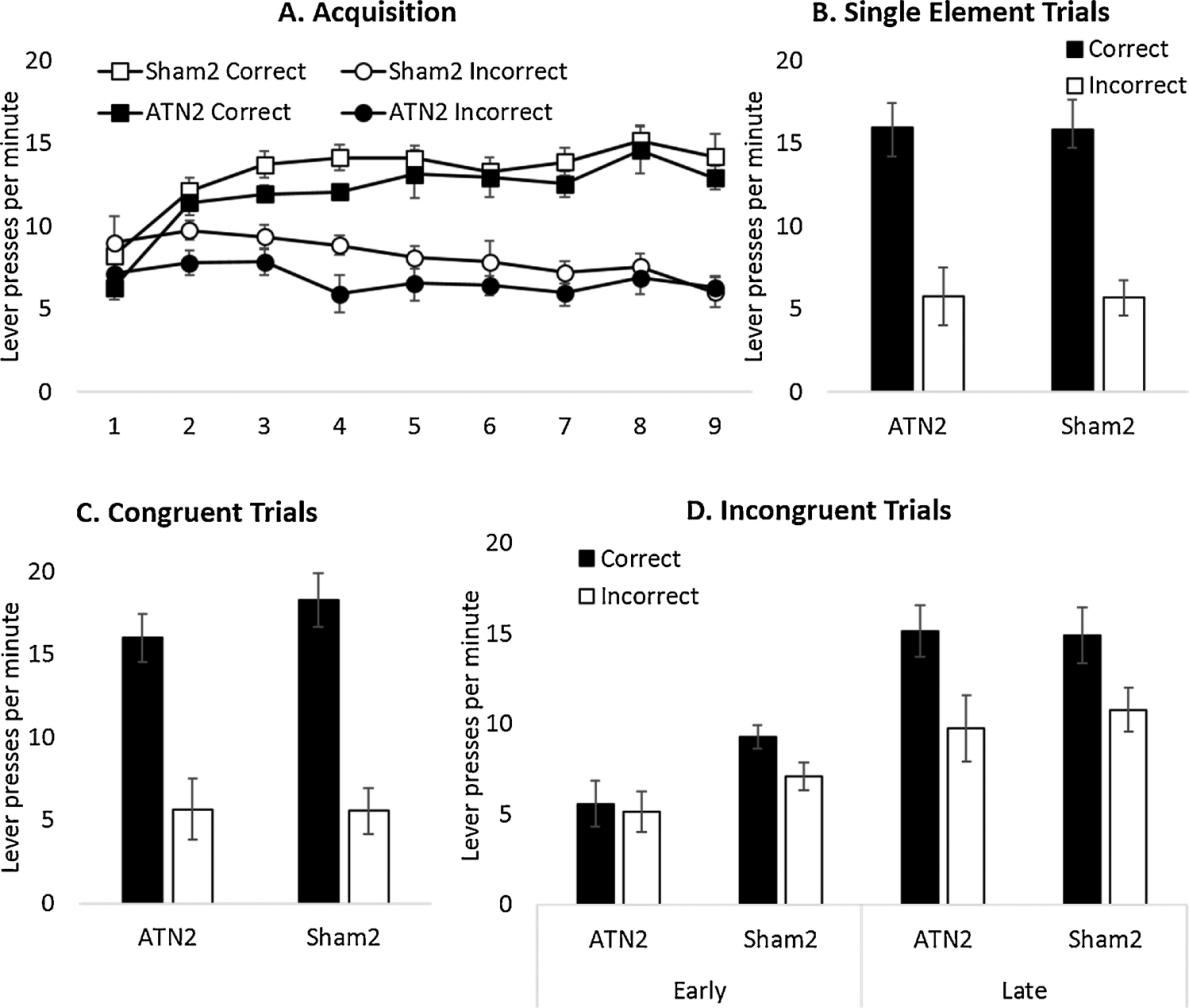
Response choice during stimulus conflict, Experiment 2. The Y axes show correct and incorrect lever presses per minute. (A) Acquisition of the conditional discriminations in blocks of two sessions. (B) In extinction, both groups showed accurate performance to the single elements used throughout acquisition. (C) In extinction, both groups showed accurate discrimination performance to congruent compounds. (D) Performance during early (the first 10 s) and late presentation of incongruent compounds. Error bars ± standard error of the mean.

Animals acquired two instrumental discriminations, one visual and one auditory, in two distinct contexts and were rewarded for pressing the correct lever with different outcomes in each context. There was no difference in the level of performance between the two discriminations (F < 1) or any interaction between Lesion and Discrimination (F < 1). Consequently, the data were analyzed collapsed across discrimination type. Both Sham2 and ATN2 groups successfully acquired the visual and auditory conditional discrimination tasks, as evidenced by a preference for the correct lever (Fig. 9A; F_1,21_ = 78.9, p < 0.001, η_p_² = 0.79) and there were no differences between the two groups (no lesion effect or interaction with block; both F < 1). Responding increased over the blocks (F_3.085,64.775_ = 3.8, p < 0.01, η_p_² = 0.154) and there was a Lever by Block interaction (F _3.085,64.775_ = 22.9, p < 0.001, η_p_² = 0.522) as correct lever press behaviour emerged over the course of training.

##### 3.2.2.2 Extinction test performance

Animals underwent extinction test sessions in which compounds of the training stimuli were presented. These compounds combined stimulus elements that dictated either the same (‘congruent’) or different (‘incongruent’) instrumental responses during initial training (Fig. 7). Animals also received trials in which the single stimulus elements were also presented. The mean response rates (correct versus incorrect) for each of the three trial types (single element, congruent and incongruent) were analysed after the four counterbalanced test sessions were combined. Thus, across the four extinction tests, there were 12 trials in total per trial type. As these tests were conducted in extinction, lever press behaviour across the full 60 s of each trial was analysed.

##### 3.2.2.3 Single stimulus elements

Both groups showed accurate conditional discrimination performance when tested on the stimulus elements acquired during training (i.e. there was as no conflict; Fig. 9B). Both groups produced more correct than incorrect responses (F_1,21_ = 54.5, p < 0.001, η_p_² = 0.722) with no effect of lesion or interaction (both F < 1).

##### 3.2.2.4 Congruent compound stimuli

Similarly, both groups produced more correct than incorrect responses (Fig. 9C) during presentation of congruent compound stimuli (F_1,21_ = 56.2, p < 0.001, η_p_² = 0.728), i.e., audiovisual compounds composed of single elements which during acquisition had been associated with the same response (i.e. no conflict). Again, there was no effect of lesion or interaction (both F < 1).

##### 3.2.2.5 Incongruent compound stimuli

Incongruent trials consisted of audiovisual compounds of single-elements that during training had elicited different responses in different contexts (Figs. 7, 9D). As expected, Control animals responded according to the stimulus-element that had previously been trained in that same test context and so appeared to use contextual cues to disambiguate the conflicting response information (Fig. 9D). Analysis of the early responding (first 10 s of incongruent compound stimuli presentation) revealed that test performance during response conflict was initially disrupted in the ATN2 group (Fig. 9D). ANOVA revealed no effect of Lever (F_1,21_ = 2.03, p = 0.17, η_p_² = 0.088) or Lever by Group interaction (F < 1). There was, however, a main effect of Group (F_1,21_ = 4.46, p < 0.05, η_p_² = 0.175) as the ATN2 group showed overall lower levels of responding during the initial presentation of conflicting cues (Fig. 9D). However, during the remaining 50 s of stimulus presentation, the ATN2 group, like the control group, responded in a context-appropriate manner and responded more on the lever associated with the test context. ANOVA confirmed an effect of Lever (F_1,21_ = 25.4, p < 0.001, η_p_² = 0.548) but no interaction with lesion or main effect of lesion (F < 1).

## 4 Discussion

Three complementary experiments examined the effects of lesions in the anterior thalamic nuclei on tests of discrimination learning with the aim of isolating the potential contribution of these nuclei to distinct aspects of behavioural flexibility. Consequently, the animals were challenged with tests designed to tax dissociable cognitive processes encompassing reversals, strategy shifts, response conflict, and the use of higher-order rules to guide behaviour (Table 1). The rationale stemmed from the extensive prefrontal and anterior cingulate connections that these nuclei possess [[7], [8], [9], [10]], raising the question of how these thalamic nuclei might contribute to nonspatial functions associated with these diverse frontal regions.

In Experiment 1B, which consisted of a series of discriminations in an automated chamber, the ATN1 group effectively learnt the discriminations but overall made more errors in the initial sessions. Although this learning deficit could not be isolated to a particular condition, there was evidence that the ATN1 group initially struggled to move from a visual to a response-based strategy when the previously correct stimulus (a light) was paired with the incorrect lever, i.e., they perseverated with the incorrect light stimulus. In the second experiment, the ATN1 rats were able to learn a spatial problem (swim to the left or right side) but made more errors in the reversal condition, in which the escape platform was now on the other side of the apparatus. The same rats then successfully solved a visual discrimination and its reversal in the swim tank. Finally, anterior thalamic damage did not appear to impact on the animals’ ability to use local contextual information to disambiguate conflicting cue information, although there was evidence that the lesion animals’ performance was reduced during the initial presentation of conflicting cues.

The current findings accord with previous evidence that simple discrimination learning is unaffected by lesions in the anterior thalamic nuclei. Spared learning has previously been reported for visual stimuli on a computer screen [42], visual cues in a swim tank [43,44] and for combinations of simple auditory and visual stimuli in an operant chamber [45]. Similarly, rats with anterior thalamic lesions were able to learn right turn/left turn discriminations in mazes when allocentric cues did not predict the correct choice [46,47]. As here (Experiment 2), rats with anterior thalamic lesions can also learn conditional rules that require both visual and response (left or right lever) discriminations [42], as well as other conditional problems that involve visual, auditory, thermal, and texture discriminations [48,49]. In contrast, a deficit was seen for a digging discrimination task that involved either olfactory or texture cues [19]. With this one exception, the overall pattern is that anterior thalamic lesions standardly spare discrimination learning, as long as the task does not involve distinguishing allocentric spatial cues [44,48]. This spared ability to acquire a wide range of discriminations contrasts with the effects of damage to the medial dorsal thalamic nucleus, which more readily impairs discrimination learning [49]. Such dissociations further underscore the heterogeneity of function within the limbic thalamus [50,51].

Both Experiments 1B and 1C incorporated tests of reversal learning, albeit with different response requirements (lever pressing versus swimming in the water tank). Previous anterior thalamic lesion studies have typically reported spared reversal learning. Examples include visual discriminations on a computer screen as well as a reversal of a visuospatial conditional rule that involved both a visual and response (left or right lever) discrimination [42], turning left or right [46], or distinguishing the right from the left side of a chamber [19]. In contrast, lesions of the medial dorsal thalamic nucleus, orbitofrontal cortex and prefrontal cortex can cause reversal deficits [[26], [27], [28], [29],^42^,[52], [53], [54], [55]], highlighting a qualitative difference between these adjacent thalamic nuclei and their respective cortical interactions. While there was evidence in Experiment 1C of an left/right reversal deficit in the water tank, it is possible that the change in contingencies may have encouraged the control rats to use heading direction to solve the problem, information that should be disrupted by anterior thalamic damage [[56], [57], [58]]. Thus, this deficit may, therefore, reflect impoverished use of spatial information rather than a general problem with reversal learning. The lack of an effect of the lesions on the response reversal task in the operant-based task (Experiment 1B) is consistent with this proposal: the critical difference between the two tasks is the water tank task placed additional navigational demands on the animals.

When required to change from one stimulus dimension to another, the rats with anterior thalamic lesions showed some evidence of initially perseverating to the previously correct visual stimulus as the distribution of errors in the first session differed from controls (Experiment 1B; Fig. 5C), while in the water tank experiment there was no evidence that the lesion animals struggled when switching between different stimulus dimensions. Previous studies using similar tasks with rats have established that such switches depend on the integrity of the prefrontal cortex and medial dorsal thalamic nucleus, but not the retrosplenial cortex [24,31,32,40]. The effects associated with prefrontal or medial dorsal thalamic damage are far more profound than those in the present study as they retarded overall learning rates. Consequently, the largely null effect found here on tests of switching indicates that the functions of the anterior thalamic nuclei are not closely aligned with those of the medial prefrontal cortex or, more specifically, the prelimbic cortex. At the same time, this transient switch effect after anterior thalamic damage appears in contrast with the apparent failure of rats with anterior thalamic lesions to form an attentional set [19], yet rapidly acquire a new discrimination involving a previously irrelevant stimulus dimension. This latter pattern would appear to predict that the switches in the current set of Experiments (1B and 1C) would also be facilitated by anterior thalamic lesions, yet this was not the case. There are, however, a number of critical differences in the tasks. Perhaps most importantly, in the present study the animals could not receive a series of new discriminations within the same domain as the choice of stimuli was restricted, e.g., right or left stimulus light in an operant box. Consequently, an attentional set could not be established in the present study. As control animals could not form an attentional set under the current testing regime, these animals were not disadvantaged when contingencies changed and, as a consequence, there was no potential for facilitation in the ATN group. It would appear, therefore, that the critical determinant of whether anterior thalamic lesions facilitate switching is the degree to which the stimulus dimension has been established as an unreliable predictor of reinforcement over multiple successive discriminations [19].

The final experiment examined the impact of anterior thalamic nuclei lesions on a rat analogue of the Stroop task that taxes behavioural flexibility in response to conflicting cue information as well as the use of higher-order rules to guide goal-directed behaviour; functions that are closely aligned to frontal cortex. Previous evidence has shown that the task is sensitive to prelimbic cortex damage, with lesion rats failing to use contextual information to disambiguate conflicting cue information [34,38]. However, as with Experiment 1B and 1C, anterior thalamic nuclei lesions did not reproduce the pattern of results associated with prelimbic damage, since the anterior thalamic nuclei lesion animals were able to use contextual information to disambiguate conflicting cue information and respond in a context-appropriate manner during the critical incongruent test trials. That lesions in the anterior thalamic nuclei spared performance on this task is unlikely to be due to ineffectiveness of the lesions, as these same animals were subsequently found to disrupt intradimensional set-shifting but, paradoxically, facilitate extradimensional set-shifting [19]. This dissociation serves to highlight how behavioural flexibility is not a unitary construct, and, consequently there is a need to consider different cognitive processes engaged by tasks classically associated with frontal cortex.

Furthermore, closer inspection of the test data did reveal that anterior thalamic lesions had an impact on task performance during the initial presentation of incongruent (i.e. conflicting) cues. During the first 10 s of stimulus presentation the lesion animals made overall fewer responses, but this effect was transient in that overall performance was unaffected by the lesion. This pattern of results is reminiscent of what is found after anterior cingulate cortex lesions, which similarly impair choice performance during the early stages of cue presentation; an impairment from which anterior cingulate, like anterior thalamic nuclei, lesion animals subsequently recover [34]. This transient impairment in choice performance on this task has been ascribed to the role of the anterior cingulate cortex in the detection of response conflict or error monitoring [34]. The implication of the current findings is that this property of the anterior cingulate cortex may depend on interactions with the anterior thalamic nuclei.

## 5 Conclusions

In summary, the current set of experiments assessed the impact of anterior thalamic nuclei damage on dissociable aspects of behaviourally flexibility known to depend on distinct sites within the rat frontal cortices. The motivation for the study came from the need to understand the potential functional significance of the dense interconnections between the anterior thalamic nuclei and these frontal areas. A clear and consistent finding was that anterior thalamic lesions did not reproduce the effects of medial prefrontal or, more specifically, prelimbic cortex damage on any of the tasks employed. Although the lesions did produce a transient impairment on the strategy-shift experiment (Experiment 1B), this deficit was mild and, perhaps more importantly, non-selective and did not reflect an inability to shift between high-order relationships, as has repeatedly been shown to be the case after medial prefrontal cortex lesions in rats [24,[30], [31], [32],^35^]. The findings from the water tank tasks underscore this observation. Furthermore, the reversal deficit in Experiment 1C was specific to spatial information indicating that the anterior thalamic nuclei do not generally contribute to reversal learning, a function classically associated with the orbitofrontal cortex. The findings from Experiment 2 do, however, indicate that the anterior thalamic nuclei may be functionally aligned with the anterior cingulate cortex, as the profile of performance on the ‘Stroop’ analogue reproduced the effects of lesions in the anterior cingulate cortex [34]. This concordance in the behavioural effects of lesions in these two sites has previously been found on tests of intradimensional set-shifting [19,59]. The demonstration that lesions in these two interconnected sites can produce analogous profiles of performance on tests of behavioural flexibility is novel. Consequently, a goal of future work will be to test the generality of these findings. A further aim will be to use disconnection procedures to determine the precise role that interactions between the anterior thalamic nuclei and anterior cingulate cortex play in the processing of non-spatial information.
